# Book Reviews

**Published:** 1815-05

**Authors:** 


### CRITICAL ANALYSIS
### OF RECENT PUBLICATIONS
IN THE
### DIFFERENT BRANCHES OF PHYSIC, SURGERY, &c.

*Observations on the Symptoms and Treatment of the Diseased Spine, more particularly relating to the Incipient Stages; with some Remarks on the consequent Palsy.* By Thomas Copeland, Fellow of the Royal College of Surgeons, and Assistant-Surgeon to the Westminster General Dispensary. 8vo. pp. 114. Callow, 1815.

Mr. Copeland's object in this treatise is to demonstrate that the formidable complaint, diseased spine, may be detected by characteristic and well-marked symptoms, long before the period of caries and incurvation. However singular it may appear, the inquiry is almost new to the profession. The early symptoms of the disease have hardly been noticed, and hitherto we have no good history of its origin and progress. When fully established in the system, indeed, we have sufficiently numerous accounts of its stubbornness and fatality. We, therefore, hope that the publication before us will not only interest the attention of practitioners on the subject, but that it will ultimately have the effect of inducing patients to consult their medical advisers at an early period of the disorder, when a more certain and favourable prognosis may be given.

After some observations on the effects of inflammation as it affects different parts; and proving, contrary to the assertion of Mr. Pott, that pressure on the spinal marrow may exist, and its consequences be detected, without any alteration tion of figure, or change of structure in the spine, being apparent; the author gives the history of the early symptoms of the disease of the spine, as follows:

"The diseased spine is to be considered as a complaint of bone or of ligament, with respect to its cure; but most of its symptoms are derived from a disturbance to the functions of the nerves, which are dependant on the spinal marrow, for their influence. There are many diseases, in which the signs and effects are in a remote part of the body from the cause which produces them; certain parts and particular structures, are, for the wisest purposes, endowed with such obscure sensation, that they may be very far advanced in disease, before any great inconvenience is produced, as long as these parts can perform the functions allotted them. The study of the sympathies of various parts with each other, in disease, and this seeming independance, and distance of the effect from its cause, of the symptom from the seat of disease, form very principal and important parts of pathology.

"When a nerve is pressed upon, with an ordinary degree of pressure, a kind of torpor, or numbness, accompanied with a sense of stricture in the part which it supplies, is produced; increase and continue the pressure, and there is loss of volition; continue and urge it yet farther, and complete paralysis takes place, loss of sensation and volition; and the most trifling injury, during this state, will produce the death and sloughing of the part. The sense of stricture, or stiffness, accompanies every stage of the compression, and is an indication that the disease does not originate in the brain. This has been well shewn, by Dr. Armstrong and others, to be a symptom of paralysis, depending on pressure on the spinal marrow; as distinguished from the general relaxed state of the muscles, in paralysis from compression of the encephalon.

"To produce this compression on the spinal marrow, there must be swelling of the intervertebral substance, or of the coverings of the medulla spinalis, or derangement of the situation of the vertebræ with respect to each other; or, lastly, some of those tumours, or other causes, which have been enumerated in the preceding chapter; and I need not illustrate by examples, how very long the knee joint, and other parts of analogous structure, will continue in a thickened state, and how often they are recovered from that state, without advancing to caries, or suppuration. I shall, hereafter, endeavour to lay down the mode of discovering the local symptoms of this stage of the complaint.

"From these two circumstances then, which may be distinguished as the *local* and the *remote* effects, are all the phenomena which Mr. Pott and others, both before and after him, have ascribed to the diseased spine, to be deduced; and if these phenomena had been more fully investigated, and traced to their source, we should long ago have been able to discover, that the most constant and the most striking of them, do, and must necessarily exist, long before any curvature or caries has taken place, and are the usual forerunners of it. I say curvature or caries, for, although though a deformity of the spine, of the description to which I allude, always supposes caries of the body of the vertebræ, instances of a very considerable degree of caries, without any curvature at all, are by no means uncommon, particularly in the neck and the loins, from the horizontal direction of the spinous processes of those vertebræ; and I have a preparation of the dorsal vertebræ, in which, the intervertebral substance is wholly removed, and the vertebræ anchylosed, without there having been any elevation of the bent spinous processes, or distortion of the form of the spine; yet this case was accompanied by paralysis."

In most cases, especially when the disease is in the superior dorsal vertebræ, the author regards a commencing paralysis of the abdominal muscles as the great characteric circumstance and symptom.

"It is surprising how very early in the disease this symptom may be detected, when the attention is directed to it. It is sometimes described as an oppression of breathing, tightness of the stomach, a band tied round the belly, torpor of the abdomen, and by other expressions in different patients. It produces costiveness, retention of urine in a more advanced stage; in short, in whatever of these symptoms you examine it, some function of the abdominal muscles is recognized to be impeded. No author who has mentioned the disease, has omitted this symptom under some name or other, although it has never, I believe, been fully explained."

Some judicious observations occur on the mode of discovering affections of the spine, before the period of incurvation. The affected part is often ascertained by its greater susceptibility to the touch, and to the stimulus of heat. The author has mentioned a simple but ingenious method of applying the test of heat by means of a sponge wrung out of hot water, and carried down the spine; while passing over the part where the disease is going on, it frequently occasions an acute degree of pain.

In the treatment of the disease, Mr. Copeland considers caustic issues as less efficacious than we had been taught to believe by Mr. Pott. Complete repose in a horizontal position is the grand remedy; blisters and leeches are useful, and in some instances the issues may be used with advantage. These being the chief means of cure, particular symptoms will be combated as they occur, according to the judgment of the practitioner. Upon the whole, we have been interested in this treatise, which we consider well calculated to awaken attention to the subject. The art and aim of the surgeon are most usefully and honourably exerted in arresting disease, before it becomes incurable, or renders an operation unavoidable.

*Welsh*

*Welsh Botanology.—Part 1st. A Systematic Catalogue of the Native Plants of the Isle of Anglesea, in Latin, English, and Welsh.—Part 2d. An Alphabetical Catalogue of the Welsh Names of Vegetables, rendered into Latin and English; with some Account of the Qualities, economical or medicinal, of the most remarkable.* By HUGH DAVIES, F.L.S. Marchant. 1813. 8vo. pp. 256.

THE readers of the works of Pennant, of English Botany, of Turner and Dillwyn's Botanist's Guide, and the Linnæan Transactions, are too well acquainted with the merits of the rector of Aber, not to promise themselves much information from any work written by him on botany or zoology. We have examined his Flora Monensis with attention, and do not hesitate to recommend it as a valuable addition to botanical knowledge, and as peculiarly useful to all lovers of botany who travel into Wales. The following specimen will enable the reader to form a tolerable idea of the manner in which it is executed.

"77. VERBASCUM. Mullein. *Pannog*.

"1. V. *Thapsus*. Great Mullein. High Taper. *Tewbannog*. *Sincyn y melinydd*. vii. viii.

"2. V. *Phoeniceum*. Purple Mullein. *Pannogbrithgoch*.

"In a scattered old fence, on the right hand from Beaumares to the alms-house in 1803. In the following year it nearly covered acres of ground in an adjoining field. I have never seen it in a garden in the neighbourhood. vii. viii."

The latter half of the volume, in Welsh, consists of an introduction to the Linnæan system of botany, and an alphabetical catalogue of the British names of plants, "each explained by the classical generic name, and by the addition of the specific when proper, and the commonly received English name; and to each species, most remarkable for its qualities, either economical or medicinal, is subjoined an account of its uses or virtues, with the manner of preparing it, and its proper dose; all taken from the best writers on those subjects, intended particularly for the use of such of my countrymen as are so distantly situated, or so circumstanced, as not to be able, on every occasion, to apply to professional men." In executing this part of the work, he speaks of "great assistance having been obtained from the ancient manuscripts of the celebrated Meddygon (physicians of) Myddfai, the history of whom," he says, "is truly curious and interesting. The first of this family that we hear of was Rhiwallon, who lived in the beginning of the 13th century. He had three sons, Cadwgan, Gruffydd, and Einion, who were brought up to the same profession. They

 were were patronised by Rhys Grug, a prince of South Wales, about 1230, who bestowed upon them a situation, lands, and privileges, at a place called Myddfai in Caermarthenshire, that they might, without molestation or interruption, attend to the study of their profession, and administer relief to such as applied to them. It is very remarkable that their descendants pursued the same science, and practised at Myddfai, till the beginning of the eighteenth century. The last of the family did not practise, and died about 1740. Mr. Owen, in his Cambrian Biography, tells us that Rhiwallon and his sons drew up a full account of the practice of physic as then known to them; and that the original manuscript is still preserved in the Welsh Charity School in London, of which there are several old copies."

If any of our correspondents should find any facts in the medical part of Welsh Botanology, or in the manuscripts of the Meddygon Myddfai, with which the medical world is unacquainted, we shall be happy to be favoured with their communications.

We cannot conclude without expressing our hope that our author will favour us with a third part, consisting of an enumeration of all the plants which he has observed in his excursions into the mountainous region of Caernarvonshire, and the adjoining counties.

---

### *Medico-Chirurgical Transactions, Volume the Fifth.*

THE variety of subjects contained in these Transactions has obliged us to procure assistance from different gentlemen best acquainted with each, which must be our apology for the irregularity with which the articles are offered to our readers.

*History of a Tubercular Eruption of a Syphilitic Appearance, but Curable without Mercury;* by T. BATEMAN, M.D. F.L.S. &c.

This case presents us with one of those numerous instances in which the attempt at representing local diseases by the pencil not only fails, but may lead us into error. In the title it is called a tubercular disease, and for such it might have passed, had there been no coloured engraving to illustrate it. Excepting that it is an eruption, we know of nothing that it possesses in common with syphilis. Whilst this strange ambiguity prevails, it will indeed, as Dr. Bateman observes, be "difficult, if not impracticable, to delineate and describe intelligibly all the varieties of eruptions which assume a character resembling that of syphilitic affections tions of the skin, but which originate, nevertheless, from the operation of mercury, or from some cachectic condition or conditions, not yet well understood; but the more this subject is investigated, the more the number of eruptions, actually found to be syphilitic, will probably be circumscribed. From the mere external character, unconnected with any history of the disease, discrimination is often impossible; but when the uniformity both of the external character and of the progress and concomitant symptoms, as in the eruption here described, is remarkable, it seems important to endeavour to fix them by the united means of the pen and the pencil. I have no other apology to offer for presenting this brief memoir to the Society."

In this instance, however, external character, if we may depend on the plate, was not likely to mislead any experienced surgeon.

Mr. T. Forster, of Guy's Hospital, has stated a case of Bubonocele, requiring a second operation five days after the first. The patient was sixty-three years of age, and ultimately recovered.

A case of Diabetes, in which opium produced an abatement of the disease, is communicated by Mr. Money, of the General Infirmary at Northampton. The dose was increased from one grain to twenty-four grains in the twenty-four hours, and very little inconvenience resulted from this large quantity. The patient, finding himself better, was discharged at his own request, and has not since been heard of; so that the only inference to be drawn from this case is, that during the use of opium, the symptoms of the disease were considerably relieved, which corresponds with our own experience; but we do not remember to have seen a single instance, in which the complaint was completely cured by the employment of opium, although we have seen it freely exhibited.

*On the Muscæ Volitantes of Nervous Persons.* By James Ware, Esq. F.R.S.

Three cases of this disease are given in full detail. It is observable, that, although essentially the same, they all differ in some particulars; and it is probable no two are to be found precisely similar. The first was characterised by a great number of small yellow moats dancing before every object to which the patient directed her eyes, their magnitude varied with the distance. If the eyes were exposed to a strong light, the air appeared full of small particles of quicksilver, in perpetual motion; among which were three or four darker coloured than the rest, which were as large as small pease, and seemed to turn round on their axis: sometimes they appeared twisted together in irregular figures, sometimes like the flue that is swept from bed-rooms. If she looked at the sky for two or three minutes, and then turned from it, her eyes flashed fire for the same space of time; looking at the sun, it appeared to be multiplied into four or five, which soon turned green, and were visible though she shut her eyes. The affection was found, on minute examination, to exist only in one eye. Twenty-five years after the commencement of the malady, they were still occasionally perceived.

Another patient complained of a considerable number of intersecting moats or beams which floated continually before his eyes, but particularly his right. Sometimes they were nearly spherical, sometimes like long knotted lines, and occasionally like a series of spherical knots, varying in number, magnitude, and opacity, sometimes undulatory, and perceived even when the eye-lids are closed. When objects were viewed at a distance, they appeared as if seen through a pane of glass sprinkled with water. He had never suffered from head-ach or pain in the eyes, though at the commencement had violent heat in them. He was short-sighted. After twelve years, he was seen by Mr. Ware a second time, when he retained the sight of both eyes, though the moats occasionally appeared in a bright light. He continued short-sighted, and used a concave glass.

In a third, several dark-coloured moats appeared before the eye, more plainly perceived when she went from one bright light to another, the intermediate space being dark; as, for instance, when she removed from looking through one window in a room to look at another. When first noticed they were seen in a bright light, but after a short time in the shade, though becoming much plainer and more numerous when the light was strong. When the lids were shut, dark spots were seen before both eyes, but not in lines till the lids were opened. She suffered no pain in the eyes; was very nervous, her spirits easily agitated, and had continually a rumbling noise in the ears. After several years the moats had not wholly disappeared. At the latter period of the complaint, she discovered an inability to read with the left eye, which was formerly short-sighted. On examination, the scene was found reversed; it had become presbyoptic, and the patient saw with a *convex* glass 36 inches focus; focus; but, curious to relate, the right eye continued myoptic, and required a *concave* glass for distant objects.

It seems that in all these cases the patients could distinguish the most minute objects, notwithstanding the confusion of the various appearances before the eyes. The pupils were dilatable: in the first and last of them, considerable nervous affection accompanied; in the two last there was short-sightedness; in the last this symptom was unaccountably changed for presbyopia in one eye, while the other was oppositely affected.

It has commonly been supposed that these moats are certain symptoms either of an incipient cataract or incipient gutta serena; and their occasional appearance with these disorders confirms the apprehension. To remove this opinion, Mr. Ware refers to the cases related in the Memoir, and to a brief description of cataract and gutta serena, which he has subjoined: from this account we select the following particulars, though they must be well known to every practitioner acquainted with diseases of the eyes.

The appearance of the cataract is *rarely sudden.* It begins by a confusion in seeing small objects, as though they were covered by a mist, which does not appear in *detached points*, but extends universally. Vision is more distinct in a dim than in a strong light, from the greater dilatation of the pupil.

After extensive inquiry, Mr. Ware cannot discover that the patients had been affected with muscæ volitantes, unless the opacity has been preceded by internal inflammation of the eye, or great muscular debility. The internal inflammation, if fully sufficient to occasion such *an alteration in the structure of the choroides*, as may, by making more or less of unequal pressure on the retina, excite the sensation of moats above described. In these, as in the cases which have been preceded by nervous debility, the moats are more perceptible when the light is strong; and where the sight is wholly extinguished, the appearance of moats ceases with it.

The diagnosis of gutta serena, given in this memoir, seems hardly to be borne out by experience. Are there no cases of this affection in its complete form, wherein the motion of the iris is preserved? When complete, Mr. W. observes, "the pupil is in general much dilated, though it may be hindered by different circumstances, particularly by the adhesion of the posterior part of the iris to the capsule of the crystalline humour. But, whether the pupil be contracted or dilated in size, it is always unchangeably fixed both in a weak and strong light; and this forms the characteristic teristic difference between the gutta serena, so alarming and too often destructive to the sight, and that comparatively harmless affection of the eye which occasions muscæ volitantes. However numerous these moats may be, and however distressing to the persons who behold them, if the power of the pupil to dilate and contract in different degrees of light remain perfect, and if the eye be able to distinguish minute characters as accurately as it did before the moats were perceived, it may be safely inferred, not only that the optic nerve retains its due degree of sensibility, but that the choroides is uninjured, since the *iris is a continuation of the membrane*, and cannot be acted upon if in a state of disease, *or if the connection between the retina and choroides be not perfect.*"

This is, certainly, a very useful paper, and particularly desireable, as containing consolatory information from an experienced practitioner on a complaint so frequently beyond the reach of art. At the same time we are obliged to confess, that we are by no means satisfied with the anatomical remarks at the conclusion of our last quotation.

*Account of an Epidemic Fever, which occurred at Gibraltar, in the Years* 1804, 1810, *and* 1813, *taken from Official Documents, Military and Medical, and from the Communications of Joseph D. A. Gilpin, M.D. Deputy-Inspector-General of Hospitals.*

Whatever relates to the doctrine of contagion is important, and to the present history the most serious respect should be paid, on account of the manner in which the inquiry is conducted, the means of information possessed by the reporters, the recency of the events, and the return of the epidemic three times in the space of ten years. We shall first offer the general remarks given at the close of the paper, afterwards our own, and then point out the few particulars in which we conceive the report deficient.

"The most obvious and important remarks, arising out of the preceding documents, are—

"I. The belief of the principal medical officers in the infectious nature of this epidemic.

"II. That the prevalence of it, like that of the plague, is limited to a particular season of the year. The season at Gibraltar, is the months of September, October, and November. The number of deaths in December is considerably above the average of the rest of the year, but they are chiefly cases in which the seizures must have been in the preceding months; for it appears from Mr. Fraser's table, that the number of new cases diminished rapidly even in November.

"III. The

"III. The various degrees of mortality in the sickly years, as compared to each other, and as compared to the years of ordinary health, are exhibited in the tables. In the year 1804, the proportion of annual mortality taken on the average strength of the garrison, was 1 in 5.4. The whole number of deaths in this year was 548, of which only 44 occurred in the nine months exempt from the epidemic. In the year 1810, the proportional annual mortality on the average strength of the garrison was 1 in 16.1. This was more equally diffused through the year, imputable, no doubt, to the presence of those regiments which had served in Walcheren. The whole number of deaths was 307, of which 187 occurred in the nine months which are not liable to the epidemic. In 1813, the proportional annual mortality on the average strength of the garrison was 1 in 10·6. The whole number of deaths was 326, of which 260 occurred in the three epidemic months.

"IV. It appears, from the reports of mortality in the three years of ordinary health, that in the three months to which the epidemic is incident, the mortality is not greater than in the other months of the year.

"V. That the average mortality of the years of ordinary health is 1 in 48.4. This is a rate of mortality considerably greater than what takes place in the same class of subjects in England; for, by the returns of population in 1811, the rate of mortality there in all ages was 1 in 49. And it appears by calculations, instituted with a view to ascertain the value of lives in granting annuities, that the mortality of persons in the prime of life is about one half of what it is on the whole population of all ages. Is this difference owing merely to climate, or in part also to some peculiar circumstances in the situation of this garrison?"

To the first remark we should reply, why have we not the report of those medical officers who do not believe in the contagious property of the disease?

2dly. If the disease only exists in the months of September, October, November, and December, and if "free ventilation has a tendency to check it" (see page 316), why is not free ventilation attended to every autumn, in order to attempt at least the prevention of an epidemic which is known to be lessened by such a measure?

3dly. If the officers were affected, it must have been by receiving the effluvium from an individual, or by inhaling an unwholesome atmosphere. If the first, we should expect that all their family, or at least all who entered the chamber, would have been affected. If they were not, what should make the effluvium from an officer under an epidemic less deleterious than from a private or a townsman?

We offer these hints for the consideration of others; at the same time adding, that, if the disease is contagious at all, it it is not contagious in the manner of small-pox, measles, and scarlatina.

The next paper that claims our notice is entitled, "Further Observations on Cataract;" being a continuation of those contained in the fourth vol. of the Transactions. Mr. Travers proceeds to offer additional remarks on certain appearances, by a minute attention to which, he conceives, the various textures of cataract may be distinguished. "That a practical value attaches to these distinctions in selecting, and in the mode of conducting, the operation," must, it is presumed, be readily admitted; and, from the opportunities which the author has enjoyed of frequently inspecting cases of this disease, we entertained the hope of finding much novel and valuable information; but, after a careful perusal, we do not discover any essential improvements on what may be collected from preceding writers on the same subject. Indeed, we rather concur in opinion with some of the best authors on this disease, that the most experienced practitioners have been not unfrequently deceived in regard to the actual nature and consistence of the opake lens; and we are best pleased with Mr. Travers, when he candidly acknowledges (p. 394) "I have myself caused such disappointment, by mistaking the nature of the cataract." We shall, therefore, be excused from making any quotations in confirmation of our sentiments.

We cannot help lamenting, that, in the enumeration of the different kinds of lenticular cataract, and their respective diagnostic symptoms, (which he has at least the merit of having compressed into one point of view,) Mr. Travers has wholly overlooked one species, usually called the **black* cataract. Has his silence been the effect of want of opportunities to witness that form of the disease; or does he, with some others, altogether discredit its existence? At all events, we are sorry he has not favoured us with his ideas on the subject, as this form of the disease is the only one at all ambiguous respecting the existence of cataract, and its diagnostic distinction from amaurosis.

The following passage is so much to the purpose, and so well expressed, that we have pleasure in giving it insertion.

"The cataracts of infants and children are either fluid or flocculent; of young adults, usually of a flocculent or soft caseous consistence; of old persons, more frequently firm, caseous, or hard.

---

* For the *symptoms* of this species of cataract, we refer our readers to the last Number of this Journal, p. 311.

Analogous to this, is our observation of the texture of the healthy lens, which is semi-fluid in children, jelly-like in adults, and gum-like in old age."

Although this is the usual condition of the lens in a state of opacity, or of health, at the periods of life mentioned, and as such ought not to be lost sight of in forming our diagnosis, still we should ever bear in mind, that under diseased action the crystalline is apt to exhibit a great variety of appearance, dependant upon its consistence; and, that whatever be its nature when the opacity is once fully established, the complaint rarely alters its primary character; or, in other words, the fluid cataract never becomes, as was erroneously supposed by the ancients, of a firm consistence, nor does the solid liquify into the fluid species.

The author's directions on the mode of depressing the solid cataract, appear, from what little knowledge we have on the subject, clear, and agreeable to the description of our best writers on couching. His remarks, also, on the immediate effects of this operation, are equally just and perspicuous; and the reasons he assigns for this process being better adapted to those cases in which the capsule is opake, than when it remains transparent, are no less satisfactory. But the picture Mr. Travers draws of the unhappy consequences which almost always, according to his experience, supervene upon couching, is truly alarming, not to say frightful, and, we would willingly hope, greatly overcharged; at least, we are constrained to form this opinion, unless we deem all that has been written by Callisern, Richter, Pott, Scarpa, and the judicious Hey, as entitled to no credit. We are therefore forced to suppose that all the ill consequence of the dislocated lens pressing upon the sensible retina, or any of the highly vascular tunics of the eye described by Mr. Travers, must have occurred under some attempt at improving the operation which Mr. Travers has had frequent opportunities of witnessing, as we cannot admit that such consequences often follow, when the operation is skilfully performed. Such dreadful effects from "the opake lens being wedged in the vitreous humour," is surely a gratuitous assertion. Are there not many instances on record in which the entire crystalline has been found imbedded in this situation *many years* after the operation of couching has been *successfully* performed, and without exciting any of those formidable disorganizing symptoms which the author dreads, and describes with so much feeling and apprehension? We are therefore compelled, after comparing what Mr. Travers has written, with the experience and declarations of the most celebrated sur-

 geons, geons, to conclude, that he must have been unfortunate in his practice with the needle, and hence has become so strenuous an advocate for extraction; for we will not permit ourselves to suppose, that he can have any wish to frighten all excepting professed oculists from undertaking the cure of cataract.

After these long remarks, we must only briefly glance at the remainder of Mr. Travers's paper. We cannot help expressing our surprise that the mode of forcing the hard cataract into the anterior chamber of the eye, should be mentioned without referring to a very old record of the accident which proved the origin of the operation by extraction. We have, indeed, been told that a rival practitioner acquired his honours by reviving this operation. But it is painful to dwell on "an old tale which every school-boy knows."

The remarks on the evacuation of the contents of the soft cataract through a puncture of the cornea, are a repetition of the method previously recommended by the late Mr. Gibson, of Manchester, whose premature death must be deeply lamented by every friend to science and humanity, and to the maintenance of whose claims we should be ashamed not to evince a proper zeal. The concluding directions relative to the mode of making the incision through the cornea, and the advantages which result from forming it of a sufficient size, are, we think, a satisfactory illustration and confirmation of what is inculcated by the best writers on extraction.

Mr. Travers gives, under twelve short heads, a summary of the principal contents of his two papers on cataract. Upon the whole, though we are disposed to think favourably of this communication, yet we cannot help objecting to the dogmatical manner in which the subject is treated; and should Mr. Travers think proper, at any future period, more fully to deliver himself upon this disease, which still admits of a great deal more without fear of exhausting it, we trust he will avail himself of our hints.

*Report of the principal Natural Diseases that have prevailed among the Children of the Royal Military Asylum at Chelsea, from its first Establishment in* 1804, *to the* 1*st of January*, 1814, *including a period of Ten Years, with some Remarks thereon.* By P. Macgregor, Esq. Surgeon to the Institution, &c.

The natural diseases included in the above title are "small-pox, measles, hooping-cough, chicken-pox, &c. which are so defined, as they are known to affect most of our species once once in their life, in every country." The definition is so satisfactory, that it may seem captious to make any objection to the term. "Natural diseases," however, does not convey a distinct meaning: we should, therefore, prefer "those contagions which produce their effect in all climates, in every season, and only once in a person's life."

The object of the paper is to remove a prejudice with some, that since the introduction of cow-pox, measles, hooping-cough, and scarlatina have been more severe, especially with such as have undergone vaccination. The result of the author's observations agree with those of Drs. Willan, Adams, and Mr. H. Field, that these contagions are usually mildest in the summer months. He, therefore, seems to admit the propriety of Dr. Adams's hint,* that, instead of constantly removing children from such contagions, by which they only gain a temporary security, it might be better, whenever the epidemic is mild, to expose them, and thus acquire a permanent relief from one of the many solicitudes to which that age is exposed. A table is added, which satisfactorily proves that vaccination does not produce in the constitution a greater liability to death from any of the other contagions.

An Appendix is added, by Dr. Henry, of Manchester. By referring to Dr. Percival's tables, and also to the last registers from the collegiate church at Manchester, it would seem that the number of deaths from measles, compared with the general mortality, had considerably increased. This is further confirmed by the register of St. John's in the same town; and if the compilers of the paper had taken the trouble of comparing our London Bills of Mortality, the scale of which is more certain in proportion as it is larger, they would have found the increase much greater. A reason is, indeed, offered for the increase in the confined tables produced, but to us unsatisfactory.

The greater mortality from the measles is easily accounted for without any implication against vaccination. Since the almost entire exemption from small-pox in that class of society to whom the preservation of their offspring is the most important, a greater number of children is reared, all of whom live to be exposed to the measles. Hence the mortality from small-pox being superseded, all who formerly died of that complaint before they were exposed to the measles, should be deducted, in order to give any accuracy to the statement. We might go further, and remark, with

---

* See his "Epidemics."

Drs. Heberden* and Adams,† the greater number of infants now reared by the improved condition of the inferior subjects in the kingdom. As the latter of these writers remarks, all those who died at an early age in number beyond the present, may, probably, be deducted from the average, as it rarely happens that children take the measles before one or even two years of age. To conclude, it is impossible accurately to ascertain how many are thus reserved to pass through the measles, but it is certain that vaccination, and the improved condition of the labouring class of society, has much increased that number of late years: and that if we take the general mortality, without allowing for the above causes, we shall find the same increase in many other diseases which cannot be suspected of the most remote connection with vaccination.

---

*Expériences sur la Digestion dans l'Homme, présenteés a la premiere Classe de l'Institut de France, le* 8 *Septembre,* 1812; par A. Jenin de Montegre, Medicin de la Faculté de Paris, Redacteur-General de la Gazette de Santé; *suivies du Rapport des Commissaires Nommées par l'Institut.* 8vo. pp. 55. Paris, 1814.

Notwithstanding the numerous experiments made by Reaumur, Spallanzani, Steevens, Gosse, and other physiologists, to ascertain the nature of digestion, the subject is still involved in a considerable degree of obscurity. Doubt yet prevails respecting the precise quality and operation of the gastric juice, its existence is even disputed, and the doctrine of putrefaction and of fermentation has still some advocates. Some physiologists contend, that the gastric juice alone is the solvent of alimentary matter, whilst others suppose a mixture of saliva with that fluid essential; others again insist on the presence of bile; and the spleen is also considered by some as assisting in the important process.

A short account of Dr. Montegre's experiments was presented by us some time ago, in our department for intelligence. We have since been favoured by that gentleman with the treatise now before us, which contains a complete account of his progress in this interesting inquiry. He possesses, it seems, the faculty of spontaneous vomiting; and thus enjoys a rich opportunity for making discoveries on the subject of digestion. By exercising this rare power when fasting, he is enabled to obtain gastric juice, and he

* Philosophical Transactions.

† Inquiry into the Laws of Epidemics.

can produce, without much effort, the food which he has swallowed at every period or stage of digestion. The object which he proposed in the inquiry was to determine what is the nature of the gastric juice, what action it performs in digestion, and, what change the aliment undergoes in the stomach. As the detail of the experiments would occupy too much space for our limits, we must be content with giving the conclusions which the author has drawn from the facts related.

It appears then, first, that the gastric juice, or the fluid which is almost always found more or less abundantly in the stomach fasting, is nothing else than saliva.

Secondly, that we cannot, consequently, regard the gastric juice as a solvent *sui generis*, of which the particular properties render it fit to prevent the putrefaction of animal substances, and still less to effect a complete digestion independently of the action of the stomach.

Thirdly, that the acidity frequently present in that juice is caused by the changes which the saliva has experienced from the stomach, changes similar to those that the alimentary matters undergo.

Fourthly, that the passage of alimentary matters to the acid state is a natural result of the action of the stomach upon them, and ought necessarily, for intensity, to depend on their chemical composition, which more or less facilitates this acidity, and in the individual dispositions of the stomach itself.

Fifthly, that the saliva and gastric juice, destined themselves to be digested, are chiefly useful in separating and liquifying the solid aliment, and communicating by their mixture the first degree of animalisation.

To these conclusions, which the author conceives are fairly deduced from the facts which he has observed, he adds, as a simple probability, waiting till additional facts can demonstrate it incontestibly, that the action of the stomach in digestion is reduced to a vital and elective absorption. The absorbent vessels, by virtue of their particular sensibility, seizing upon certain portions of the alimentary matter, abandoning others, in the same manner as it happens in the whole of the alimentary conduit, where the nutritive particles are continually seized by the vessels in the middle of the fœces destined to be ejected.

The author has not yet been able to ascertain the exact nature of the acid so frequently present in the gastric juice, but is disposed to consider it as acetic acid. He announces his intention of losing no opportunity in prosecuting the enquiry.

In

In the report of Messrs. Berthollet, Cuvier, and Thenard, which accompanies the treatise, these philosophers admit that the experiments of Dr. De Montegre have thrown light on the nature of the gastric fluid; but they do not accord with his explanation of digestion depending on a simple separation produced by an elective absorption.

The cause of digestion then still remains to be ascertained; and the discovery of Dr. De Montegre amounts to this, that saliva and gastric juice are similar in their nature and action on alimentary matter. Food exposed to saliva under similar circumstances, will be acted upon precisely the same, as if subjected to gastric juice. But we may be allowed to doubt whether the stomach of the ingenious author, which so readily affords him gastric liquor or food during the process of digestion, is in a healthy condition, and whether the fluid he obtained was, in fact, pure gastric juice. If the two fluids are precisely identical, it is probable that gastric fluid is not a secretion peculiar to the stomach, but merely saliva which is continually passing into it from the mouth, and sometimes, as is well known, in considerable quantity. At all events, we consider the enquiry quite open; much remains to be ascertained; we cannot rest satisfied with experiments made on one individual. We therefore hope, that Dr. De Montegre will pursue his researches, and that physiologists of our own country may be induced to take up this very interesting subject.

---

